# A pragmatic behavior-based habit index for adherence to nebulized treatments among adults with cystic fibrosis

**DOI:** 10.2147/PPA.S186417

**Published:** 2019-02-13

**Authors:** Zhe Hui Hoo, Martin J Wildman, Michael J Campbell, Stephen J Walters, Benjamin Gardner

**Affiliations:** 1 School of Health and Related Research (ScHARR), University of Sheffield, Sheffield, UK, z.hoo@sheffield.ac.uk; 2 Sheffield Adult Cystic Fibrosis Centre, Northern General Hospital, Sheffield, UK, z.hoo@sheffield.ac.uk; 3 Department of Psychology, Institute of Psychiatry, Psychology and Neuroscience (IoPPN) King’s College London, London, UK

**Keywords:** cystic fibrosis, medication adherence, nebulizers and vaporizers, habits

## Abstract

**Background:**

Habit, a psychological process that automatically generates urges to perform a behavior in associated settings, is potentially an important determinant of medication adherence. Habit is challenging to measure because, as a psychological construct, it cannot be directly observed. We describe a method of using routinely available objective adherence data from electronic data capture (EDC) to generate a behavior-based index of adherence habit and demonstrate how this index can be applied.

**Methods to generate the habit index:**

Our proposed habit index is a “frequency in context” measure. It estimates habit as a multiplicative product of behavior frequency (generated from weekly percentage adherence) and context stability (inferred from time of nebulizer use). Although different timescales can be used, we chose to generate weekly habit scores since we believe that this is the most granular level at which context stability can be reasonably calculated.

**An application of the habit index:**

A hallmark of habit is to predict future behavior, hence we used time series method to cross-correlate the habit index with nebulizer adherence in the subsequent week among 123 adults with cystic fibrosis (52, 42.3% female; median age 25 years) over a median duration of 153 weeks (IQR 74–198 weeks). The mean cross-correlation coefficient (*R*) between the habit index and subsequent adherence was 0.40 (95% CI 0.36–0.44). Adjusting for current adherence, the unstandardized regression coefficient (*B*) for the habit index was 0.30 (95% CI −1.04 to 1.65).

**Conclusion:**

We have described a pragmatic method to infer “habit” from adherence data routinely captured with EDC and provided proof-of-principle evidence regarding the feasibility of this concept. The continuous stream of data from EDC allows the habit index to unobtrusively assess “habit” at various time points over prolonged periods, and hence the habit index may be applicable in habit formation studies.

## Introduction

Low medication adherence in long-term conditions is associated with worse health outcomes and higher health care cost,^1^–^3^ yet only around 50% of all medications are taken as recommended.^4^,^5^ In cystic fibrosis (CF) – a genetic long-term condition predominantly affecting the lungs and gastrointestinal tract – high adherence is associated with better health outcomes in terms of reduced pulmonary exacerbation risk, more stable lung function, reduced hospitalization risk, and reduced health care costs.^6^–^10^ Efforts to improve medication adherence are therefore important to improve health outcomes, but various systematic reviews have shown that most adherence interventions are not efficacious.^11^,^12^ Even efficacious interventions only have inconsistent or short-term effects, with improved adherence not maintained beyond the intervention period.^11^,^12^

Interventions that effectively initiate behavior change may not necessarily sustain that change.^13^,^14^ A potential mechanism for sustaining behavior change is habit formation. Within psychology, the term “habit” describes a non-conscious process by which a situational cue (eg, time of day) automatically generates an impulse toward enacting a behavior (eg, taking medication), based on learned associations between the cue and the behavior.^15^ Habit associations are acquired when a specific behavior is repeated consistently in a specific setting.^15^ As habit develops, cognitive control over behavior shifts from reflective to automatic processes, such that encountering a contextual cue is sufficient to elicit the associated behavior, with little deliberation or forethought.^15^ Behavior maintained by ongoing effortful self-control and conscious motivation would be susceptible to disruption when self-control is reduced; for example, during times of stress or if there is a need to devote resources to other cognitively effortful tasks.^15^ However, automatic control of habitual behavior reduces the dependency on conscious attention or deliberative processes, such that habitual behavior should persist even when attention or conscious motivation wane.^16^ Indeed, studies in various long-term conditions have highlighted the strong association between habit strength and medication adherence.^17^–^23^ A recent meta-analysis of 771 trials also found medication adherence interventions that included habit formation were more effective than those that did not.^24^ Studies examining the association between medication adherence and habit rely on accurate measurements of habit, but measuring habit is challenging.

As a psychological process, habit cannot be directly observed, and so existing measures infer habit only indirectly. While lab-based tests exist eg, the Implicit Association Test,^25^ in the real-world habit is measured by self-report, predominantly using one of two types of measures. One involves self-reporting markers of habit. For example, the Self-Report Habit Index (SRHI)^26^ and its derivative, the Self-Report Behavioral Automaticity Index (SRBAI),^27^ infer habit from reflection on the “symptoms” of habitual responding, such as lack of awareness, conscious intention, or control. Another involves reporting the frequency of previous behavior in a stable context to infer habit as a form of “frequency in context”,^28^ based on the assumption that “strong habits” have been performed frequently in stable contexts whereas “weak habits” have been performed in unstable context or performed less frequently.^29^ The development of self-reported habit measures has enabled the study of habit and allowed the role of habit to be better understood.^30^ For example, some of the seminal papers in habit are based on frequency in context measures.^31^,^32^

Nonetheless, the use of self-report to assess habit measures has been criticized.^33^,^34^ A think-aloud study has shown that people may misinterpret some of the SRHI items, or infer automaticity based on frequency of action (although the frequent behavior may have actually required considerable deliberative planning or cognitive effort).^35^ It is also argued that people’s recollection of behavior and experience is unreliable.^33^ A potential method of enhancing the frequency in context measure might be to bypass the subjectivity of self-report by using objective data for inferring behavior frequency and context stability.

Unlike habit, the behavior of using preventative inhaled therapy among adults with CF can be directly captured using electronic data capture (EDC), which is generally considered the “gold standard” method to capture adherence data.^36^ In CF, tamper-proof intelligent nebulizer systems (I-neb^®^, Philips, Amsterdam, the Netherlands) which provide date-and time-stamped data for every dose of nebulized medication are routinely available.^37^ The I-neb^®^ is a third-generation data-logging adaptive aerosol delivery system designed to optimize inhalation technique by directing flow and depth of inhalation, providing positive feedback signals to guide user, and only delivering aerosol when an inhalation of sufficient quality is detected.^38^ By routinely and accurately logging every episode of medication use (including date and time of use), both elements of a “frequency in context” measure, ie, “behavior frequency” and “context stability” (with time as a potential cue for nebulizer use, since previous studies have demonstrated that time of day is a useful cue for medication taking),^39^ are being captured. Thus, it may be possible to infer habitual responses (to time cues) using these objective data.

In addition to providing a truer reflection for both the actual frequency and time context of nebulizer use, the continuous stream of data from EDC also makes it feasible to unobtrusively assess “habit” at various time points over prolonged periods. EDC data are routinely available without any additional effort from adults with CF (ie, data are automatically captured as long as nebulizer is being used). That means the habit index generated from EDC data would impose no additional burden on study participants, so would be more “participant-friendly” than administering multiple self-report items.

In this paper, we aim to describe a pragmatic method of inferring “habit” from objective nebulizer adherence data and to demonstrate how this behavior-based index of adherence habit might be applied, using a predictive design to explore its relationship with subsequent adherence.

### A description of our proposed behavior-based habit index

This habit index is based on the format of a “frequency in context” measure. That means the proposed index is calculated by multiplying “behavior frequency” with “context stability”. Frequency in context measures is based on the theory that repetition in a consistent context leads to habit formation, and so more frequent repetitions (in a consistent context) and greater context stability (given equal number of repetitions) mean strongest likelihood of habit having formed, ie, represent strongest habit.

The method described in this paper would generate a weekly index in a linear 1–7 scale. This index could also be calculated using different time periods (eg, fortnightly or monthly) or transformed linearly into different scales (eg, 0–100).

### Calculating “behavior frequency” using adherence data captured by EDC

A behavior frequency score can be simply generated by converting weekly percentage adherence into a linear scale from 1.00 (adherence 0%) to 2.65 (adherence ≥100%). Using a 1–2.65 scale for “behavior frequency” score would result in a 1–7 habit index scale because the habit index is a product of “behavior frequency” and “context stability” (1^2^=1; 2.65^2^=7). This linear scale can be altered depending on preference. For example, a 1–100 habit index scale can be generated by using 1–10 scales for “behavior frequency” and “context stability”.

### Calculating “context stability” using adherence data captured by EDC

**Figure 1:**
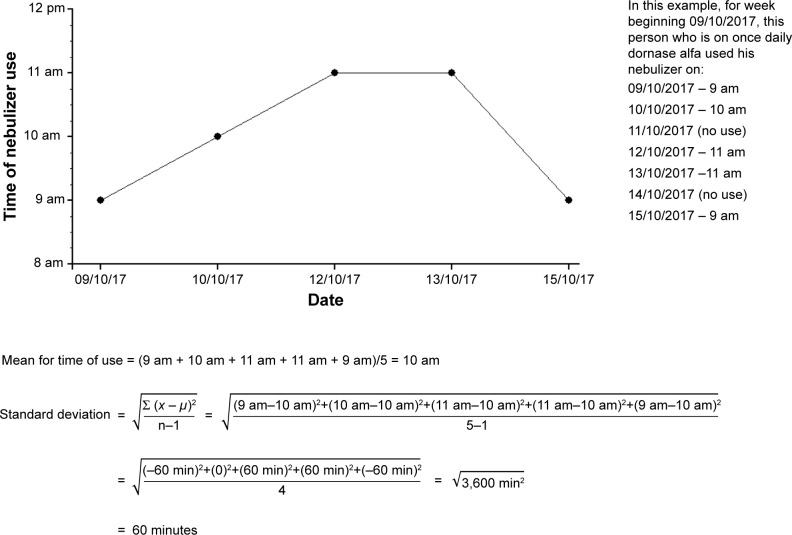
An example to illustrate the calculation of SD as a measure of variability for the time of nebulizer use.

The actual time of nebulizer use can be determined from date- and time-stamped EDC adherence data to infer “context”. Stability/variability in context can be calculated as SD. An example of calculating variability in time of use is provided in Figure 1.

In calculating the variability for time of use, it is important to consider that adults with CF may use their inhaled preventative therapies in two separate sessions because inhaled antibiotics, eg, colistin and tobramycin, are meant for twice daily use, with the UK CF Trust recommending a minimum interval of 6 hours between doses.^40^ Both treatment sessions should be considered separately because behavior for one session may be discordant to the behavior in the other session (eg, adherence during “evening” sessions may be better compared to adherence during “morning” sessions^41^ or vice versa). In considering the “morning” and “evening” sessions, using midnight–mid-day and mid-day–midnight scales is likely to be problematic. Many young people will go to bed after midnight, partly because the timing of melatonin release shifts to a later time during adolescence.^42^ It is not uncommon for these “night owls” to use their inhaled therapy after midnight (eg, after returning from a night out). For example, a person may use his inhaled antibiotic at 10 am, and take the final dose of the day just before bed, which may on occasions be 1 am or 2 am. Using a 05:00–16:59 scale for the morning session and a 17:00–04:59 scale for the evening session may better reflect nebulizer use in the real world. Concordant sessions should be used to calculate variability in time of use, ie, morning sessions should be considered separate to evening sessions. Overall variability can then be calculated by applying weights relative to the number of sessions with ≥1 nebulizer use per week.

**Figure 2:**
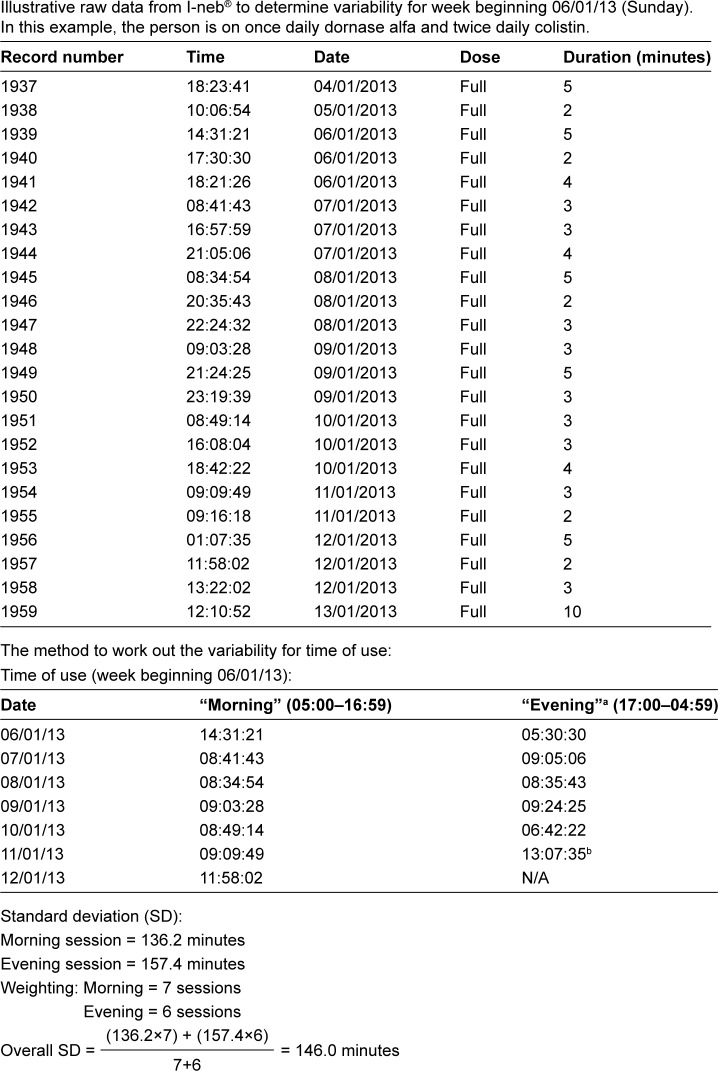
An example to illustrate the calculation of SD for time of use in someone using nebulizer over two sessions. **Notes:** ^a^Evening times were transformed into morning times for accuracy of calculation, otherwise nebulizer taken after midnight may cause spuriously large SD depending on the software used. For example, in someone who uses nebuliser at 23:45, 23:55, 00:15 and 23:52, the difference between 00:15 and 23:55 could be miscalculated as 23 hours and 40 minutes (instead of 20 minutes) if the date was not taken into account. ^b^Although this treatment was taken after midnight, it is still part of the evening session treatment for 11/01/2013. **Abbreviation:** NA, not available.

In calculating the variability for time of use, it is also important to consider that adults with CF may be using more than one dose of nebulizer in a single treatment session. For example, someone using nebulized colistin twice daily and dornase alfa once daily would be using a dose of colistin and a dose of dornase alfa in one of the two treatment sessions. The time between both doses of nebulizer within the same session could be affected by other treatment routines, eg, chest physiotherapy. Therefore, only the time for the first nebulizer use of each session should be considered in calculating the variability in time of use. An example to illustrate that the calculation of variability in time of nebulizer use is based on SD is provided in Figure 2.

Following calculation of SD for time of nebulizer use as a variability measure, the context stability scores for time of nebulizer use can be generated by converting the variability measure into a linear scale from:

1. 1.00 (variability in time of use ≥180 minutes, ie, the maximum value), to
2. 2.65 (variability in time of use =0, ie, the minimum value).

If the nebulizer was not used at all in a week, the maximum variability value for time of use should be assigned (ie, 180 minutes), so that the minimum context stability score is generated to indicate minimal context stability. The same “penalty” should be applied if nebulizer use was too infrequent to calculate variability, since at least two separate values are needed to calculate SD.

In the example presented in Figure 2, the context stability score based on SD as a measure of variability for time of nebulizer use would be:

$$=2.65-1.65\left(\frac{Variability}{180}\right)=2.65-1.65\left(\frac{146.0}{180}\right)=1.31$$

### Calculating the proposed behavior-based habit index using adherence data captured by EDC

The proposed habit index (ranging from 1 to 7) is a product of “behavior frequency” (ranging from 1 to 2.65) and “context stability” (ranging from 1 to 2.65).

In the example presented in Figure 2, 20/21 doses of nebulizer were used during week beginning 06/01/2013. The adherence of 95.2% translates to “behavior frequency” score of 2.57. Therefore, the habit index for week beginning 06/01/2013 would equate to 3.4 (ie, 2.57×1.31) for time of nebulizer use as the context.

### An example application of the habit index

#### A hallmark of habit–habit strength should predict the performance of future behavior

A potential role of the proposed habit index is to predict future adherence, since a hallmark of habit measures’ predictive validity is the relationship between habit strength and performance of future behaviors. For example, Ouellette and Wood demonstrated that self-reported “frequency in context” (ie, past behavior × context stability) is a stronger predictor of future television watching and recycling behaviors, compared to past behavior alone.^43^

For medication adherence, prolonged repetition of the behavior is required for health benefits to be realized. Empirical evidence suggests that health behaviors can be highly variable over time.^44^–^47^ Likewise, psychological processes are also inherently variable over time.^48^,^49^ Thus, there is merit in studying the relationship between the behavior of using medication and psychological processes over the long term, to understand how this relationship changes over time. Adherence data that are routinely and accurately logged by EDC provide the opportunity to study the dynamic variability of the proposed habit index and adherence at a more granular level by using time series methods. Time series analysis refers to statistical methods used to analyze time-ordered serial measurements, and it can be used to predict future behaviors, explain characteristics of behaviors, and understand factors influencing the behaviors.^50^,^51^ We therefore set out to determine the cross-correlation between the proposed behavior-based habit index and subsequent nebulizer adherence.

## Methods

### Design and setting

Data were drawn from a single-center retrospective observational study. All adults with CF diagnosed according to the UK CF Trust criteria^52^ in Sheffield, UK, aged ≥16 years, and using I-neb^®^ as part of their routine treatment were included, except those with lung transplantation or on ivacaftor. Both lung transplantation and ivacaftor have transformative effects on lung health,^53^–^55^ such that their treatment requirements may no longer represent that of a typical adult with CF.^56^,^57^

All I-neb^®^ data for a calendar year period were excluded if there was no nebulizer use for >24 consecutive weeks or for >90% of the time period on I-neb^®^ (eg, 24 weeks of I-neb^®^ data available but nebulizer was only used in two of those weeks). This data exclusion is to avoid spurious cross-correlation between the behavior-based habit index (which will be at the minimum score of “1” if I-neb^®^ was not being used) and nebulizer adherence (which will also be at the minimum score of “0” if I-neb^®^ was not being used). This study was approved by the NHS Health Research Authority (IRAS number: 210313). This study was carried out in accordance with the principles of the Declaration of Helsinki. All participants provided written informed consent for the analysis of their routinely collected clinical and adherence data.

### Data collection and processing

Clinical data including age, gender at birth, %FEV_1_, and nebulizer prescription details were extracted by two investigators independently reviewing paper notes and electronic records. Nebulizer adherence data were downloaded from I-neb^®^. Adherence was calculated as a percentage of total nebulizer doses taken against the agreed dose between clinicians and adults with CF. Based on this method of quantifying adherence, adherence levels can vary from 0% to >100% (due to potential nebulizer overuse), with higher adherence being more desirable although nebulizer adherence >100% may not be optimum (this may vary with the medications – hypertonic saline may be beneficial if used more frequently whereas antibiotics may cause toxicity if used substantially more frequently than the prescribed doses).

Weekly behavior-based habit index, which can vary from 1 (ie, weakest habit) to 7 (ie, strongest habit), for each study participant was generated from I-neb^®^ data with methods as described in the “a description of our proposed behavior-based habit index” section by using a pre-programmed Microsoft Excel v.2010 (Microsoft) spreadsheet. “Context stability” was inferred from time of nebulizer use, and variability in context was calculated using SD.

### Data analysis

Analyses were performed using SPSS v24 (IBM Corporation). Each weekly habit index from each study participant was cross-correlated with percentage adherence at the subsequent week using a linear regression model with time-ordered habit index as a single covariate and adherence as the dependent variable (ie, habit index at Week 1 correlated with adherence at Week 2, habit index at Week 2 correlated with adherence at Week 3, and so on). This method models the trends of weekly habit index and adherence as linear functions of time^49^ but does not account for autocorrelation in the data,^58^ meaning that the resultant cross-correlation coefficient will conflate both the genuine (changes in adherence that are actually related to prior changes in habit index) and spurious (independent changes of both habit index and adherence with time) associations between these two time-ordered variables.

Therefore, multiple regression was also performed for each study participant using both time-ordered habit index and current adherence as the covariates, with subsequent percentage adherence as the dependent variable. Using both habit index and percentage adherence as the covariates achieved two important functions. First, autocorrelation is removed from adherence data, ie, the genuine association between the habit index and subsequent adherence can be determined. Second, it controls for present adherence. This is crucial because nebulizer adherence among adults with CF is an ongoing behavior (ie, behaviors that have been performed many times and are still continuing). Correlation of a psychological construct with an ongoing behavior will conflate both the effect of the psychological construct on behavior and the effect of behavior on the psychological construct.^59^ By controlling for present behavior, bias from the effects of behavior on the psychological construct is mitigated; that is, the resultant correlation coefficient will be a purer measure of the effect of the psychological construct on behavior.^59^

Following time series analyses of individual data, the individual results were aggregated and analyzed at a group level. Appropriate descriptive statistics were generated, with both the effect sizes and CI for the habit index–adherence associations being reported. Both the correlation coefficients (*R*) for the habit index–adherence association from univariate linear regression and unstandardized coefficients (*B*) for the habit index from multiple regression were also stratified according to stability of adherence. This is to explore whether habit index–adherence associations were influenced by different adherence patterns. The three a priori adherence patterns of interest are 1) people with consistently low adherence (ie, all 3-monthly adherence sections of ≤25%), 2) people with consistently high adherence (ie, all 3-monthly adherence sections of >75%), and 3) everyone else with varying adherence. The use of 3-monthly adherence sections and the ≤25%/>75% thresholds to understand whether adherence is changing over time are based on an adherence clustering algorithm that was previously published.^60^

## Results

**Figure 3:**
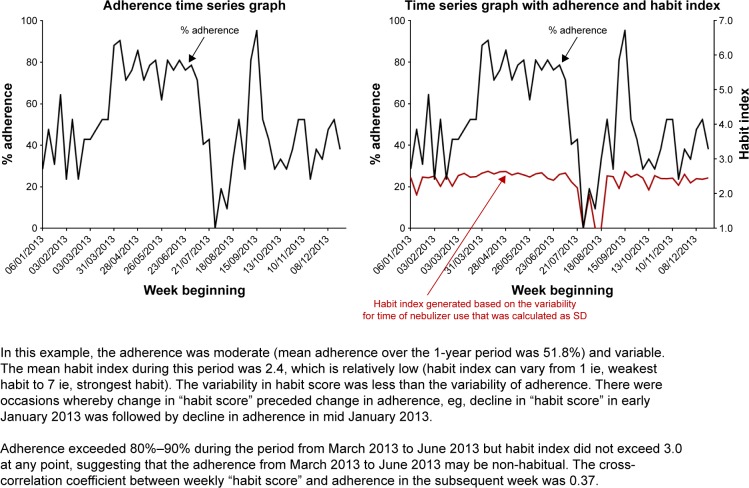
Illustrative habit index (generated from time of nebulizer use) and adherence time series graphs.

This analysis included 123 out of 126 adults with I-neb^®^ data. Data for three adults who only had minimal nebulizer use were excluded (Person 1: adherence 0.2% over 39 weeks; Person 2: adherence 8.7% over 47 weeks; Person 3: adherence 2.3% over 22 weeks). Of the 123 included adults, 52 (42.3%) were females. The median age of this cohort was 25 years (IQR 19–31 years) and median %FEV_1_ was 74.0% (IQR 54.9%–87.5%). Median adherence of the cohort was 47.3% (IQR 26.2%–76.4%), with median data duration of 153 weeks (IQR 74–198 weeks). Most of the adults (99/123, 80.5%) had variable adherence. An example of adherence time series graph along with the habit index superimposed on the graph for a person with variable adherence is presented in Figure 3.

The mean cross-correlation coefficient between the habit index and subsequent adherence was 0.40 (95% CI 0.36–0.44) for the entire cohort (Table 1). However, those with variable adherence displayed higher mean cross-correlation coefficients (mean 0.45) compared to those with consistent adherence (mean 0.20–0.24).

By including both the habit index and current adherence as the covariates in a multiple regression, the regression coefficient (*B*) for habit index was small (mean 0.30; ie, 1 unit increase in the habit index was associated with a 0.3% increase in the subsequent week’s adherence, after controlling for current adherence). The 95% CI also included negative values (−1.04 to 1.65; ie, increase in habit index may be associated with decline in subsequent week’s adherence, after controlling for current adherence) (Table 2).

Part of the higher cross-correlation coefficient for the habit index–subsequent adherence association among people with variable adherence was due to autocorrelation in adherence data. After removing the autocorrelation in adherence data with the multiple regression analysis, those with variable adherence had the lowest regression coefficient (mean 0.08, 95% CI −1.44 to 1.60).

## Discussion

We have described a pragmatic method of inferring “habit” for the behavior of using nebulizer and demonstrated the feasibility of generating weekly habit index among a cohort of 123 adults with CF over a median period of 153 weeks. We were able to evaluate the habit index over a prolonged period because health care for people with CF is almost exclusively delivered by multidisciplinary teams through specialist CF centers, hence loss to follow-up is unlikely to occur. In addition, this is an observational study utilizing routinely collected data without burdensome additional data collection. The habit index was generated from routinely collected I-neb^®^ data, and an I-neb^®^ typically stores 3,000–4,000 datapoints.^37^

Habit is an automatic process by which behavior is contextually cued.^15^ As habit forms through associative learning during context-dependent repetitions, cognitive control of a behavior shifts from reflective to automatic processes, which reduce the dependence of habitual behaviors on conscious attention or deliberative processes.^15^ Habitual behavior should therefore persist even if attention or conscious motivation wane, ie, habit can shield new behaviors from relapse and determine behavior frequency.^15^ We provided an example of applying the habit index to study its association with subsequent nebulizer adherence. Treatment regime for a person with CF typically consists of multiple components due to the multisystem nature of CF. A similar habit index could be generated for other treatment modalities (or even medications for other long-term conditions) in which timestamped EDC data are available, such as Medication Event Monitoring System for oral medications, eg, pancreatic enzyme replacement therapy or chipped physiotherapy adjuncts. Using data from intelligent nebulizers (I-neb^®^), we found a mean cross-correlation coefficient of 0.40 between the “habit scores” and nebulizer adherence in the following week. This is comparable to the average correlation coefficient (0.39–0.53) shown in previous research between the dominant self-report habit measure (the SRHI) and a range of health behaviors.^27^,^61^

A study looking at the habit of using asthma medications found a larger correlation coefficient of 0.61 between SRHI and adherence.^18^ However, it is important to interpret these coefficients in the context of the analysis method. Previous studies have only compared habit strength against behavior using measurements at one to three time points,^18^,^27^,^61^ whereas we cross-correlated habit index against subsequent adherence at multiple time points using time series analysis. Time series methods allow the relationship between habit and behavior to be studied at a more granular level (eg, week-by-week relationships can be studied instead of just using measurements at one or two time points) and directionality of relationships to be evaluated (habit strength can be cross-correlated with subsequent adherence to understand how prior changes in habit relate to subsequent changes in adherence).^49^ Cross-correlation using time series methods for time-ordered data at multiple points will typically yield lower coefficients compared to correlation coefficients compared at only a few time points. For example, correlation of previous nebulizer adherence measured on an annual basis (ie, adherence measured at a “single time point”) against subsequent annual nebulizer adherence using the Sheffield dataset yielded correlation coefficients of 0.82 (95% CI 0.73–0.88) for 2013–2014 (n=79 pairs of measurements); 0.84 (95% CI 0.76–0.89) for 2014–2015 (n=92 pairs); and 0.85 (95% CI 0.78–0.90) for 2015–2016 (n=93 pairs). Such high correlation coefficients were obtained by comparing annual adherence despite clear year-on-year improvement of adherence from 43.6% in 2013 to 55.1% in 2016 as previously reported.^62^ In contrast, cross-correlation of time-ordered adherence data against adherence in the subsequent week (ie, lag 1 autocorrelation) among the 123 study participants included in this analysis only yielded a mean coefficient of 0.51 (95% CI 0.47–0.55).

After controlling for current behavior (by performing multiple regression of time-ordered habit index and current adherence as the covariates), the proposed habit index was found to have little effect on subsequent adherence (mean unstandardized coefficient of 0.3). This is in part because controlling for present behavior would underestimate the effects of psychological construct on subsequent behavior.^59^ The strong correlation between past and future behaviors is also well recognized.^43^,^63^,^64^ Studies using self-report measures have also tended to find higher correlation between behaviors and habit measures which included behavior frequency. For example, the validation study for SRBAI (an automaticity subscale of SRHI) found higher correlation between behavior and SRHI (which included items of behavior frequency), compared to SRBAI (which only included automaticity items).^27^ An unpublished study included both behavior frequency × context stability (BFCS) scores (a frequency in context self-report measure) and SRHI scores in a multiple regression model, and found that only BFCS scores explained significant variance in behavior.^29^

Although past behavior does predict future behavior, studying a behavior by just using previous behavior as a proxy for habit lacks explanatory value as to why the behavior was enacted or how it might change in the future. Therefore, developing an adequate habit measure remains important. By demonstrating the association between our proposed habit index and subsequent adherence using time series analysis, we have highlighted that the index can potentially track how habit changes over time. The habit index may be applicable in habit formation studies. For example, it might be used in a study investigating the development of habit to understand how long it takes to form the habit of using nebulizer, or to investigate fluctuation in habit over time.

We nonetheless acknowledge the limitations of inferring “habit” from EDC adherence data. For example, “habit” would be inferred as “absent” when a behavior was not enacted. Yet habit is not behavior, so it is possible that someone may have a habit that they are not acting on, for whatever reason.^15^ As a form of frequency in context measure, the proposed habit index also assumes a compensatory relationship between behavior frequency and context stability (ie, frequent behavior in varying settings is expected to have the same influence on habit strength as infrequent behavior in unvarying setting), but this assumption is untested. Existing EDC adherence data lack contextual information other than time of use. Any feature in the environment including (but not limited to) locations, presence of other people, and prior responses in a sequence of actions can cue a behavior.^15^ Although the exact time of day (eg, 7 am) can act as a contextual cue for medication adherence,^39^ it may be that prior action (eg, after brushing teeth in the morning) is a more relevant contextual cue to instigate nebulizer use among adults with CF. Some activities are nonetheless time sensitive (eg, people watching the 10 pm news would do so at 10 pm), while other activities (eg, waking from sleep) may recur every 24 hours, though the actual time may vary (eg, among shift workers). As technology advances, it is likely that EDC in the future will capture more contextual information. Advances in sensor technology has allowed the development of “context-aware” reminder systems to support behavior change, ie, reminders that are only triggered in the appropriate environmental settings.^65^–^67^ These sensors could potentially be used to capture extensive contextual information related to enactment of behavior, eg, setting/location in which behavior is enacted, prior actions, and presence of other people. As these technologies become ubiquitous, it may be possible to infer “habit” using a range of such contexts for different individuals (ie, tailor the “habit” measure according to idiosyncratic context cues that activate impulse toward behavior).

Regardless of the contextual information available, there is uncertainty regarding the optimal method to measure context stability. We chose to measure context stability on a weekly basis because that is the most granular level at which the habit index can be generated, and studying the habit index at the most granular level, in theory, allows earlier detection of behavior change. However, it might be that a behavior needs to be observed over a longer period of time in order to infer “stable habit” from that behavior. Non-performance of a behavior does not necessarily imply habit has changed, but missing the opportunity to enact a behavior or a slight change in routine would alter the proposed habit index if it were being inferred from behaviors over a short time duration. It is uncertain how quickly habit strength for nebulizer use among adults with CF changes over time. A previous study modeling habit formation for a new healthy eating, drinking, or exercise behavior found that the median time for habit strength (measured with 7 out of 12 items from the SRHI) to plateau was 66 days.^68^ However, there was considerable variation between individuals (range for the cohort was 18–254 days) and between behaviors (longer time for exercise behavior compared to healthy eating or drinking), though, potentially due to small sample size, the difference was not statistically significant.^68^ If variability was being measured over a fixed time period (ie, the method that we have proposed), frequency of behavior would influence the number of readings available to calculate the index of variability. For example, SD for time of nebulizer use could only be calculated from two readings if someone only used nebulizer in two sessions of that week, whereas seven readings will be available to calculate SD in someone who used nebulizer in all seven sessions of that week. SDs may be influenced by the number of readings available. If there were only a few readings, spuriously low (or high) SD values may be obtained. In the example we presented in Figure 3, there was a small increase in the habit index despite quite substantial rise in adherence, in part because variability in time of nebulizer use for that person increased when the nebulizer was being used more frequently. This might reflect that use was not habitual, or that time is not the contextual cue for habitual nebulizer use for that person, or that the method to measure variability was insufficiently sensitive and requires improvement. An alternative method to determine the “context stability” score would be to use a fixed amount of data in calculating variability (eg, calculate SD for every seven sessions of nebulizer use, regardless of when these sessions occur). However, this means the habit index could only be calculated infrequently for people with low adherence. This might not matter if habit tends not to fluctuate over short time periods. In terms of the variability index itself, different methods can be used to calculate variability including coefficient of variation (ie, ratio of the SD to the mean),^69^ “median deviation” (ie, the difference between the maximum and median value),^70^ or sigma (which signifies variation-over-time and is calculated from moving ranges).^71^ Our previous analyses found little difference between SD, “median deviation”, or sigma (data not shown). In theory, sigma accounts for time order in data dispersion^71^ and may be the most suitable index of variability for this purpose. There is also uncertainty regarding the limits for variability indices in the calculation of “context stability” scores (eg, variability in time of nebulizer use of ≥180 minutes was assumed to infer minimum “context stability”). Due to these methodological uncertainties, further exploration using a larger dataset could potentially model the fluctuation of “context stability” over time to further refine the habit index.

There are also limitations with available methodologies to evaluate a habit index inferred from EDC adherence data. There is no “gold standard” habit measure to validate the habit index. Using a “less correct” measure (eg, any of the self-report habit measures) for validation^72^ is feasible. However, it would not be possible to determine whether differences between the habit index and a self-report habit measure is due to the habit index lacking validity, or the self-report habit measure lacking validity, or a combination of both. Since the hallmark of habit measures’ predictive validity is to predict the likelihood or frequency of future behavior’s enactment,^15^ we determined the correlation between the proposed habit index and subsequent adherence. It could be argued that using the same dataset to generate and then to evaluate the habit index would inflate the association between “habit” and adherence. However, habit “at baseline” was being correlated with subsequent adherence, ie, identical data were not being correlated. It is nonetheless recognized that correlating a psychological construct with a behavior using observational data would conflate effects of behavior on the psychological construct as well as effects of the psychological construct on behavior, especially for ongoing behaviors.^59^ Controlling for current behavior (eg, by performing multiple regression of time-ordered habit index and adherence as the covariates in our analysis) helps to mitigate against bias from the effects of behavior on perception.^59^ However, we acknowledge that our analysis is not a definitive test for the proposed habit index, and further validation work is required. This is ideally carried out using an experimental design (eg, studying habit formation in randomized clinical trials whereby the construct of interest is prespecified and mediational analyses were conducted to determine whether the construct served as a vehicle of change), since this type of study yields the least ambiguous cause–effect conclusions and offers opportunities to establish causality.^59^

Developing an adequate habit measure is important to better understand the relevance of habit to medication adherence. We have described a method of inferring a habit index from routinely available objective nebulizer adherence data among adults with CF and provided an example of how the habit index can be applied to study the relationship between habit and medication adherence. There is uncertainty regarding the optimal method to infer context stability, and further validation of the proposed habit index is required. Current EDC data also lack sufficient contextual information about the behavior, although context stability can potentially be inferred more reliably with the advent of better context-sensing technology. This may be a promising avenue to further explore in terms of developing useful habit measures, since there is no method to directly measure habit impulse.

## Figures and Tables

**Table 1:** The cross-correlation coefficients (*R*) for the habit index and subsequent adherence

Adherence type	Habit index cross-correlation coefficient, mean (95% CI)
Overall cohort (n=123)	0.40 (0.36–0.44)
Adherence consistently low, ie, ≤25% (n=6)	0.24 (0.04–0.44)
Variable adherence (n=99)	0.45 (0.41–0.49)
Adherence consistently high, ie, >75% (n=18)	0.20 (0.13–0.27)

**Table 2:** The unstandardized regression coefficients (*B*)^a^ for the habit index, using time-ordered habit index and current adherence as the covariates with subsequent adherence as the dependent variable in a multiple regression

	Habit index unstandardized coefficient, mean (95% CI)
Overall (n=123)	0.30 (−1.04 to 1.65)
Adherence consistently low, ie, ≤25% (n=6)	3.03 (−9.68 to 15.76)
Variable adherence (n=99)	0.08 (−1.44 to 1.60)
Adherence consistently high, ie, >75% (n=18)	0.61 (−1.90 to 3.13)

**Note:**

a A regression coefficient of 0.30 meant that 1 unit increase in the habit index (which can vary from 1 [ie, weakest habit] to 7 [ie, strongest habit]) was associated with a 0.3% increase in the subsequent week’s adherence (which can vary from 0 to >100%).
